# DCBLD1 is associated with the integrin signaling pathway and has prognostic value in non-small cell lung and invasive breast carcinoma

**DOI:** 10.1038/s41598-021-92090-6

**Published:** 2021-06-17

**Authors:** Guillaume B. Cardin, Monique Bernard, Francis Rodier, Apostolos Christopoulos

**Affiliations:** 1 Centre de recherche du Centre hospitalier de l’université de Montréal, Montreal, QC Canada; 2 Institut du cancer de Montréal, 900 Saint-Denis, Montreal, QC H2X 0A9 Canada; 3 Département de radiologie, radio-oncologie et médecine nucléaire, Université de Montréal, Montreal, QC Canada; 4 Otolaryngology-Head and Neck Surgery Service, Centre hospitalier de l’Université de Montréal, Montreal, QC Canada

**Keywords:** Cancer, Oncology

## Abstract

Germline single nucleotide polymorphisms in the promoter region of the *DCBLD1* gene are associated with non-smoking cases of both non-small cell lung carcinoma (NSCLC) and human papillomavirus-negative head and neck cancer. However the clinical relevance and function of *DCBLD1* remain unclear. This multicenter retrospective study was designed to evaluate the prognostic value and function of *DCBLD1* in the four main solid cancers: NSCLC, invasive breast carcinoma, colorectal adenocarcinoma and prostate adenocarcinoma. We included the following cohorts: GSE81089 NSCLC, METABRIC invasive breast carcinoma, GSE14333 colorectal adenocarcinoma, GSE70770 prostate adenocarcinoma and The Cancer Genome Atlas (TCGA) Firehose Legacy cohorts of all four cancers. *DCBLD1* gene expression was associated with a worse overall survival in multivariate analyses for both NSCLC cohorts (TCGA: *P* = 0.03 and GSE81089: *P* = 0.04) and both invasive breast carcinoma cohorts (TCGA: *P* = 0.02 and METABRIC: *P* < 0.001). Patients with high *DCBLD1* expression showed an upregulation of the integrin signaling pathway in comparison to those with low *DCBLD1* expression in the TCGA NSCLC cohort (FDR = 5.16 × 10^–14^) and TCGA invasive breast carcinoma cohort (FDR = 1.94 × 10^–05^).

## Introduction

The oncologic relevance of the *DCBLD1* gene was first described in a genome-wide association study that identified a susceptibility locus in the *DCBLD1* promoter in women with never-smoking lung cancer^1^. Three subsequent studies have reported an association between single nucleotide polymorphisms (SNPs) in the *DCBLD1* promoter region and higher risks of never-smoking cancers for human papillomavirus-negative head and neck squamous cell carcinoma (HNSCC), lung cancer, and female lung adenocarcinoma (LUAD)^2–4^. SNPs in this locus were associated with higher *DCBLD1* expression in HNSCC and lung cancer^2,5^, and with overall survival in two other studies, one on female non-smoking patients with lung cancer, and another in non-small cell lung carcinoma (NSCLC)^4,6^. The prognostic value of *DCBLD1* gene expression was also demonstrated for HNSCC and LUAD by multivariate and univariate analyses respectively^2,5^. Furthermore, NSCLC xenografts in mice using A549 cells showed that knockdown of the *DCBLD1* gene greatly impaired tumor growth *in vivo*^7^.

Little is known about the function of the DCBLD1 protein, a transmembrane protein with extracellular CUB, LCCL and F5/8 type C domains^8^. These features are similar to the extracellular domain of neuropilins (NRPs), which are transmembrane proteins that bind to semaphorins and have established roles in neural axon guidance^8^. DCBLD1 may have a similar function as NRPs; DCBLD1 was shown to be upregulated in mitral cells during the olfactory learning of rat pups while co-localizing with semaphorin 4c and correlated with extracellular matrix (ECM) remodeling, suggesting a role in neural development^9^. In oncology, NRPs are involved in breast, prostate, renal and pancreatic cancers, mainly through their interactions with integrins^10–13^. By comparison, gene ontology analysis of *DCBLD1* expression in HNSCC showed a strong upregulation of the integrin signaling pathway in patients with high *DCBLD1* expression, suggesting another parallel with NRPs^2^.

The intracellular region of DCBLD1 contains seven highly conserved YxxP motifs that are involved in phosphorylation-dependent binding to the CRKL signaling protein^14^. The Abl and Fyn kinases are responsible for this phosphorylation^14,15^. Phosphoprotemic bioinformatic analysis showed that the phosphorylation of four of these YxxP sites was altered following the inhibition of EGFR and MET and following HGF and FGF2 stimulation, suggesting an association of DCBLD1 with receptor tyrosine kinases^2^. Thus far, the only study on the interactome of DCBLD1 involved proteomic analysis on HEK 293 cells transfected with a FLAG-tagged DCBLD1 plasmid^15^. With this overexpression model, the FLAG immunoprecipitate revealed that the majority of DCBLD1 interactors were adaptor proteins and proteins associated with actin dynamics^15^.

Although multiple studies have now described SNPs in the *DCBLD1* promoter region in different oncology contexts^1–7^, the role of DCBLD1 itself in cancer and its function in both normal and oncology settings remain poorly understood. The objective of this study was to evaluate the prognostic value of DCBLD1 in the four most frequent solid cancers^16^: NSCLC, breast, colorectal, and prostate cancers. Two independent cohorts were used for each cancer. We also investigated the DCBLD1 status in The Cancer Genome Atlas (TCGA) PanCancer Atlas to determine the potential oncogenic mechanism of DCBLD1.

## Results

### Patient cohorts

NSCLC, invasive breast carcinoma, colorectal adenocarcinoma and prostate adenocarcinoma were each represented by two cohorts for the analysis of *DCBLD1* gene expression and cancer outcome. Age, sex and stage distribution of these cohorts are shown in Table 1.

**Table 1:** Demographics and clinical parameters of the cohorts evaluated in the outcome analysis.

	NSCLC TCGA	NSCLC GSE81089	Breast TCGA	Breast METABRIC	Colorectal TCGA	Colorectal GSE14333	Prostate TCGA	Prostate GSE70770
Total, n	1001	199	1091	1900	372	236	496	203
Age, median (range)	67 (38–90)	69 (45–84)	58 (26–90)	61 (21–96)	66 (31–90)	67 (26–92)	61 (41–78)	62 (41–73)
**Sex, n (%)**								
Female	400 (40)	103 (52)	1079 (99)	1900 (100)	166 (45)	112 (47)	0	0
Male	601 (60)	96 (48)	12 (1)	0	206 (55)	124 (53)	496 (100)	203 (100)
**Stage, n (%)**								
1	513 (51)	115 (58)	180 (16)	475 (25)	56 (15)	45 (19)	37 (7)	11 (5)
2	279 (28)	48 (24)	618 (57)	800 (42)	134 (36)	100 (42)	339 (68)	72 (35)
3	164 (16)	33 (17)	249 (23)	115 (6)	112 (30)	91 (39)	39 (8)	107 (53)
4	33 (3)	3 (2)	20 (2)	9 (0)	52 (14)	0 (0)	81 (16)	13 (6)
NA	25 (2)	0 (0)	24 (2)	501 (26)	18 (5)	0 (0)	0 (0)	0 (0)
Follow-up, months (median)	21.5	36.4	27.8	115.6	22.1	38.1	30.5	36.7
**Tobacco use, n (%)**								
Ever smoker	885 (88)	180 (90)	–	–	–	–	–	–
Never smoker	91 (9)	19 (10)	–	–	–	–	–	–
NA	25 (2)	0	–	–	–	–	–	–
**NSCLC histology, n (%)**								
Adenocarcinoma	506 (51)	108 (54)	–	–	–	–	–	–
Squamous cell carcinoma	495 (49)	67 (34)	–	–	–	–	–	–
NA	0	24 (12)	–	–	–	–	–	–
**PAM50 subtype, n (%)**								
Luminal A	–	–	413 (38)	679 (36)	–	–	–	–
Luminal B	–	–	174 (16)	460 (24)	–	–	–	–
HER2-enriched	–	–	65 (6)	218 (11)	–	–	–	–
Basal-like	–	–	136 (12)	397 (21)	–	–	–	–
Normal-like	–	–	25 (2)	140 (7)	–	–	–	–
NA	–	–	278 (25)	6 (0)	–	–	–	–

### DCBLD1 gene expression and cancer outcome

We previously evaluated the role of the *DCBLD1* gene in association with patient outcome in HNSCC^2^. For NSCLC, this association was only tested in univariate analysis on one cohort^5^, and nothing is yet known for breast, colorectal and prostate cancers. We examined if *DCBLD1* gene expression had prognostic value in the eight cohorts of this study, using multivariate analysis with age, sex (when appropriate) and stage. The hazard ratio (HR) was based on the range of *DCBLD1* expression levels, which was analyzed as a numerical variable. A higher HR was associated with a higher risk for patients who had high *DCBLD1* gene expression. This type of analysis is less biased and more stringent than a cut-off based analysis^17^. It also shows a continuity of the risk increase through the variable distribution. Age was also analyzed as a continuous variable. Patients were not subdivided by sex for prostate and breast cancers as there were only 12 males in the TCGA cohort and no male in the Molecular Taxonomy of Breast Cancer International Consortium (METABRIC) cohort. Stages were grouped as stage 1 and 2 versus stage 3 and 4 to prevent bias from the low frequency of some stages for certain cancers. These stage groupings resulted in the smallest group to be n = 36 (for stage 3–4, NSCLC GSE81089 cohort).

**Figure 1:**
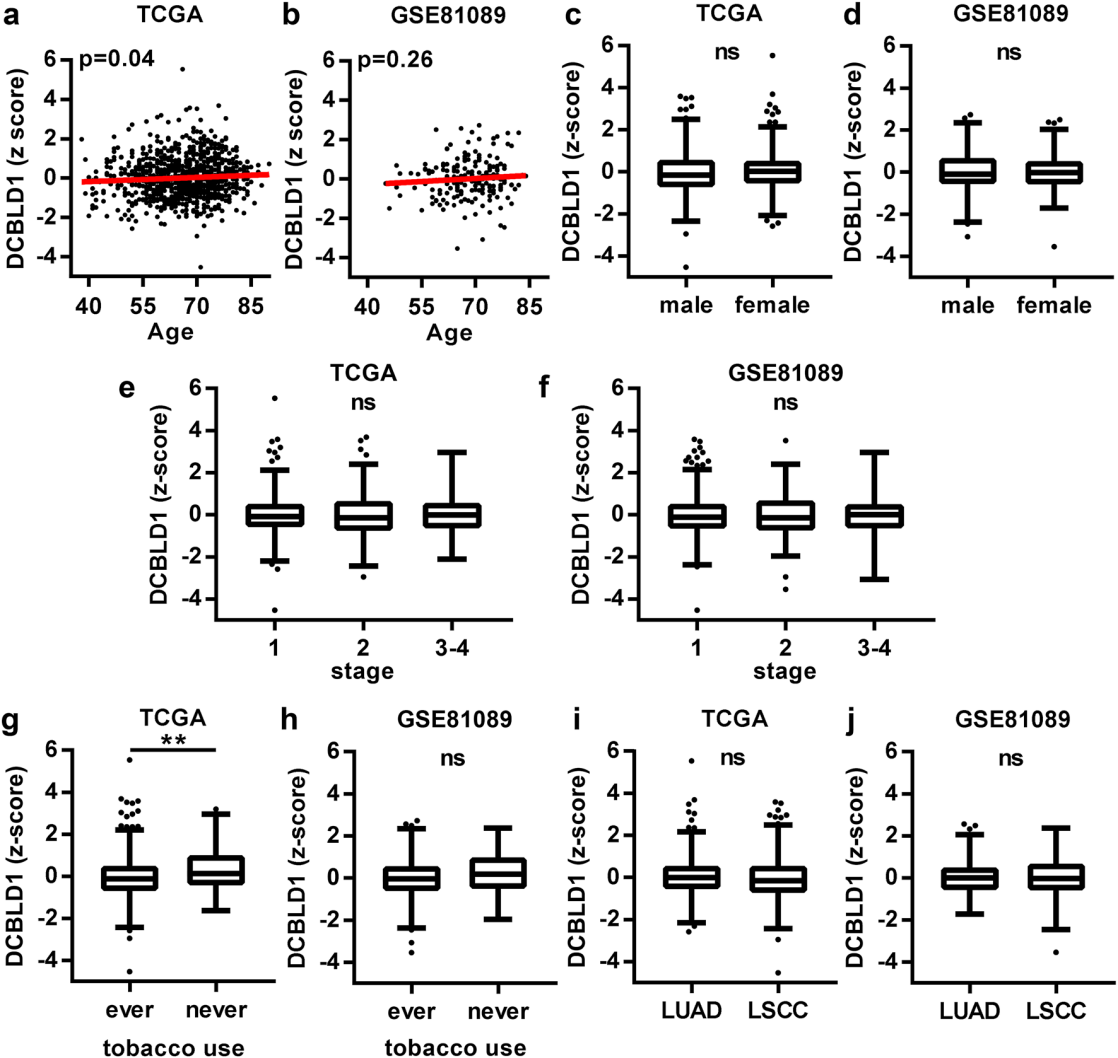
DCBLD1 in the TCGA and GSE81089 cohorts of non-small cell lung carcinoma. Comparison of DCBLD1 gene expression for age (**A**, **B**), sex (**C**, **D**), stage (**E**, **F**), tobacco use (**G**, **H**) and histology (**I**, **J**).

For NSCLC, both TCGA and GSE81089 cohorts had reproducible significant results. High *DCBLD1* gene expression and stage were significantly associated with a worst overall survival, while age and sex had no significant effect (Table 2). When the same analysis was performed without stratifying stage into two groups, and histology and smoking status were added to the model, high *DCBLD1* gene expression was again associated with a worst overall survival (Supplementary Table 1). Age (Fig. 1A,B), sex (Fig. 1C,D), stage (Fig. 1E,F), smoking history (Fig. 1G,H) and histology (Fig. 1I,J) were not reproducibly associated with *DCBLD1* gene expression for NSCLC.

**Table 2:** Multivariate Cox proportional hazards analysis of cancer outcomes.

Variable	HR (95% CI)	*P*	HR (95% CI)	*P*
**Non-small cell lung carcinoma—overall survival**				
	**TCGA**		**GSE81089**	
DCBLD1 expression (numerical)	3.03 (1.11–8.04)	0.03	3.71 (1.05–13.09)	0.04
Age (numerical)	1.79 (0.99–3.27)	0.06	1.32 (0.48–3.71)	0.59
Sex (male vs female)	1.16 (0.93–1.43)	0.17	1.35 (0.90–2.05)	0.15
Stage (3–4 vs 1–2)	1.96 (1.56–2.44)	< 0.001	2.41 (1.49–3.80)	< 0.001
**Invasive breast carcinoma—overall survival**				
	**TCGA**		**METABRIC**	
DCBLD1 expression (numerical)	10.08 (1.49–60.66)	0.02	2.20 (1.38–3.48)	< 0.001
Age (numerical)	7.98 (3.40–18.80)	< 0.001	12.27 (7.73–19.56)	< 0.001
Stage (3–4 vs 1–2)	3.10 (2.16–4.43)	< 0.001	2.28 (1.82–2.81)	< 0.001
**Colorectal adenocarcinoma—overall survival**				
	**TCGA**		**GSE14333**	
DCBLD1 expression (numerical)	2.39 (0.34–16.38)	0.38	1.40 (0.25–7.85)	0.70
Age (numerical)	9.20 (2.98–29.46)	< 0.001	0.42 (0.11–1.56)	0.42
Sex (male vs female)	1.02 (0.65–1.63)	0.93	1.00 (0.57–1.76)	1.00
Stage (3–4 vs 1–2)	3.73 (2.30–6.22)	< 0.001	3.05 (1.74–5.49)	< 0.001
**Prostate adenocarcinoma—biochemical recurrence**				
	**TCGA**		**GSE70770**	
DCBLD1 expression (numerical)	0.95 (0.11–8.49)	0.97	1.07 (0.08–16.79)	0.96
Age (numerical)	1.93 (0.51–7.51)	0.34	1.40 (0.14–16.79)	0.78
Stage (3–4 vs 1–2)	3.34 (1.99–5.57)	< 0.001	2.36 (0.78–10.15)	0.14

**Figure 2:**
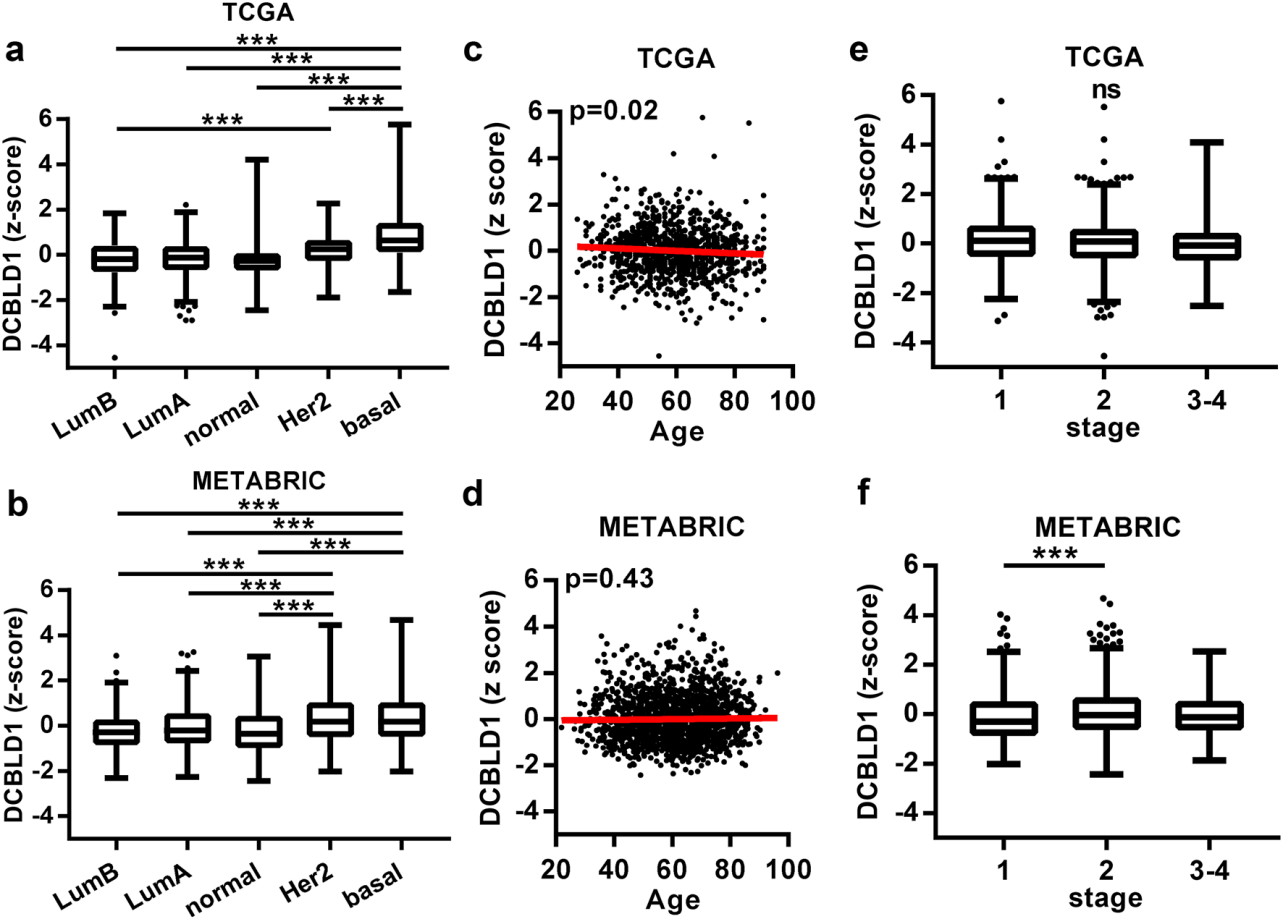
DCBLD1 in the TCGA and METABRIC cohorts of invasive breast cancer. Comparison of DCBLD1 gene expression for PAM50 subtypes (**A**, **B**), age (**C**, **D**) and stage (**E**, **F**).

For invasive breast carcinoma, the TCGA and METABRIC cohorts also showed reproducible significant results. High *DCBLD1* gene expression, age and stage were significantly associated with a worst overall survival (Table 2). Specifically, *DCBLD1* expression was associated with the PAM50 molecular subtypes in both cohorts (Fig. 2A,B). Basal-like and HER2-enriched breast cancers had significantly higher *DCBLD1* expression compared to the normal-like and luminal subtypes in the METABRIC cohort. In the TCGA cohort, the HER2-enriched subtype was not significantly different from the normal-like and luminal subtypes, but we observed lower *DCBLD1* expression in the basal-like subtype. Since PAM50 subtypes carry prognostic value^18^, this may partly explain a DCBLD1 association with survival. Indeed, adding the PAM50 parameter to the multivariate model in both cohorts lowered the association of *DCBLD1* gene expression with overall survival with *P* values of 0.05 for the TCGA cohort and 0.02 for the METABRIC cohorts (Supplementary Table 2). Age (Fig. 2C,D) and stage (Fig. 2E,F) were not reproducibly associated with *DCBLD1* gene expression for invasive breast carcinoma.

For colorectal adenocarcinoma, no association was found between DCBLD1 and overall survival in both the TCGA and the GSE14333 cohorts (Table 2). Also, no association was observed between *DCBLD1* gene expression and age, sex or stage in colorectal adenocarcinoma (Supplementary Fig. 1).

For prostate adenocarcinoma, biochemical recurrence was evaluated instead of overall survival due to the low numbers of deceased patients. Biochemical recurrence in prostate cancer is defined as a rise in the blood level of prostate-specific antigen following surgery, and it precedes clinical disease recurrence^19^. Only 10 of 496 participants were deceased in the TCGA cohort (median follow-up: 30.5 years). No association was found between DCBLD1 and biochemical recurrence in both the TCGA and GSE70770 cohorts (Table 2). Also, no association was observed between *DCBLD1* gene expression and age or stage in prostate adenocarcinoma (Supplementary Fig. 2).

### DCBLD1 expression in tumor tissues

**Figure 3:**
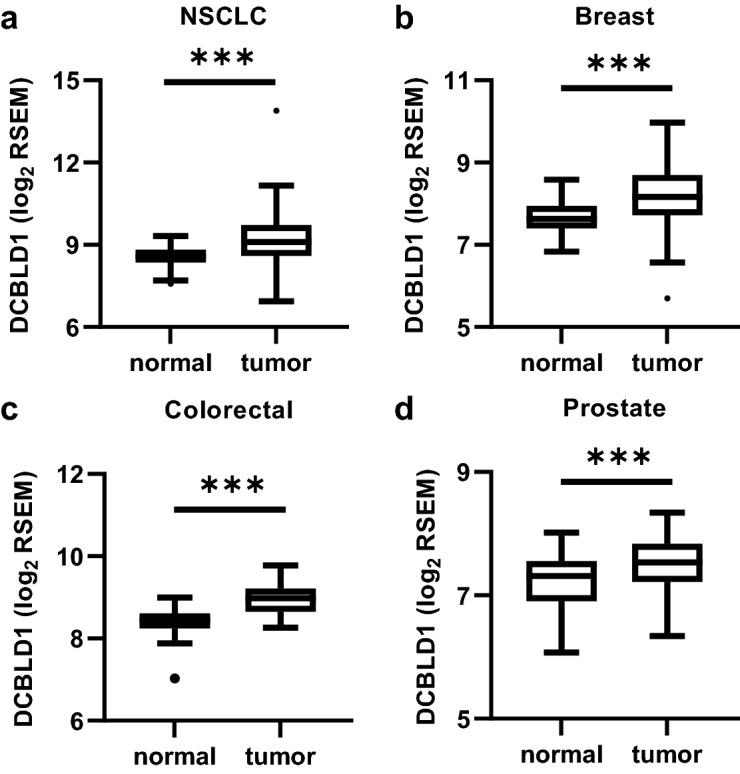
Evaluation of DCBLD1 expression in tumor tissue. Comparison of DCBLD1 gene expression in normal tissue adjacent to the tumor and tumor tissue for NSCLC (**A**), invasive breast carcinoma (**B**), colorectal adenocarcinoma (**C**) and prostate adenocarcinoma (**D**) TCGA cohorts.

*DCBLD1* gene expression was investigated in paired tumor tissue and normal adjacent tissue in the NSCLC (n = 108), invasive breast carcinoma (n = 111), colorectal adenocarcinoma (n = 32) and prostate adenocarcinoma (n = 52) TCGA cohorts (Fig. 3). Only participants with RNAseq results for both tissues were considered for this analysis. Statistically significant higher *DCBLD1* in tumor tissue was observed for all four cancers with median of 1.47 fold for NSCLC, 1.54 fold for invasive breast carcinoma, 1.39 fold for colorectal adenocarcinoma and 1.25 fold for prostate adenocarcinoma.

### *DCBLD1* mutations and copy number alterations in cancer

*DCBLD1* was investigated within the TCGA PanCancer Atlas. Occurrence of mutations was evaluated, resulting in only 109 patients of 10,953 (1.0%) harboring mutations in the DCBLD1 protein coding region, and no single mutation was present in more than four total cases (0.04%) (Supplementary Fig. 3). The only cancer with over 3% of mutations was uterine cancer with 33 cases out of 517 (6.4%) (Supplementary Fig. 4). Copy number alterations were rare with only 36 patients (0.3%) showing amplification and 77 patients (0.7%) showing a deep deletion of *DCBLD1* (Supplementary Fig. 5).

### Upregulated and downregulated genes and pathways in patients with high DCBLD1 expression

To understand the implications of high *DCBLD1* expression, we compared the RNA-seq gene expression profiles of 50 patients with the highest *DCBLD1* expression versus 50 participants with the lowest *DCBLD1* expression in each of the four TCGA cohorts independently. We evaluated pathway enrichment in the four cohorts using the PANTHER pathway database (Table 3). Patients with high *DCBLD1* expression had strong upregulation of the integrin signaling pathway in comparison to patients with low *DCBLD1* expression for both NSCLC and invasive breast cancer. No pathway was upregulated in the colorectal and prostate cancer cohorts. Also, no pathway was downregulated in patients with high *DCBLD1* expression in all four cohorts.

**Table 3:** Significantly upregulated pathways for DCBLD1 high expression patients in the four TCGA cohorts.

Cancer	Upregulated PANTHER pathway	FDR
Non-small cell lung carcinoma	Integrin signaling pathway	5.16 × 10^–14^
	Inflammation mediated by chemokine and cytokine signaling pathway	9.33 × 10^–04^
Invasive breast carcinoma	Integrin signaling pathway	1.94 × 10^–05^
Colorectal adenocarcinoma	No significantly upregulated pathway	–
Prostate adenocarcinoma	No significantly upregulated pathway	–

**Figure 4:**
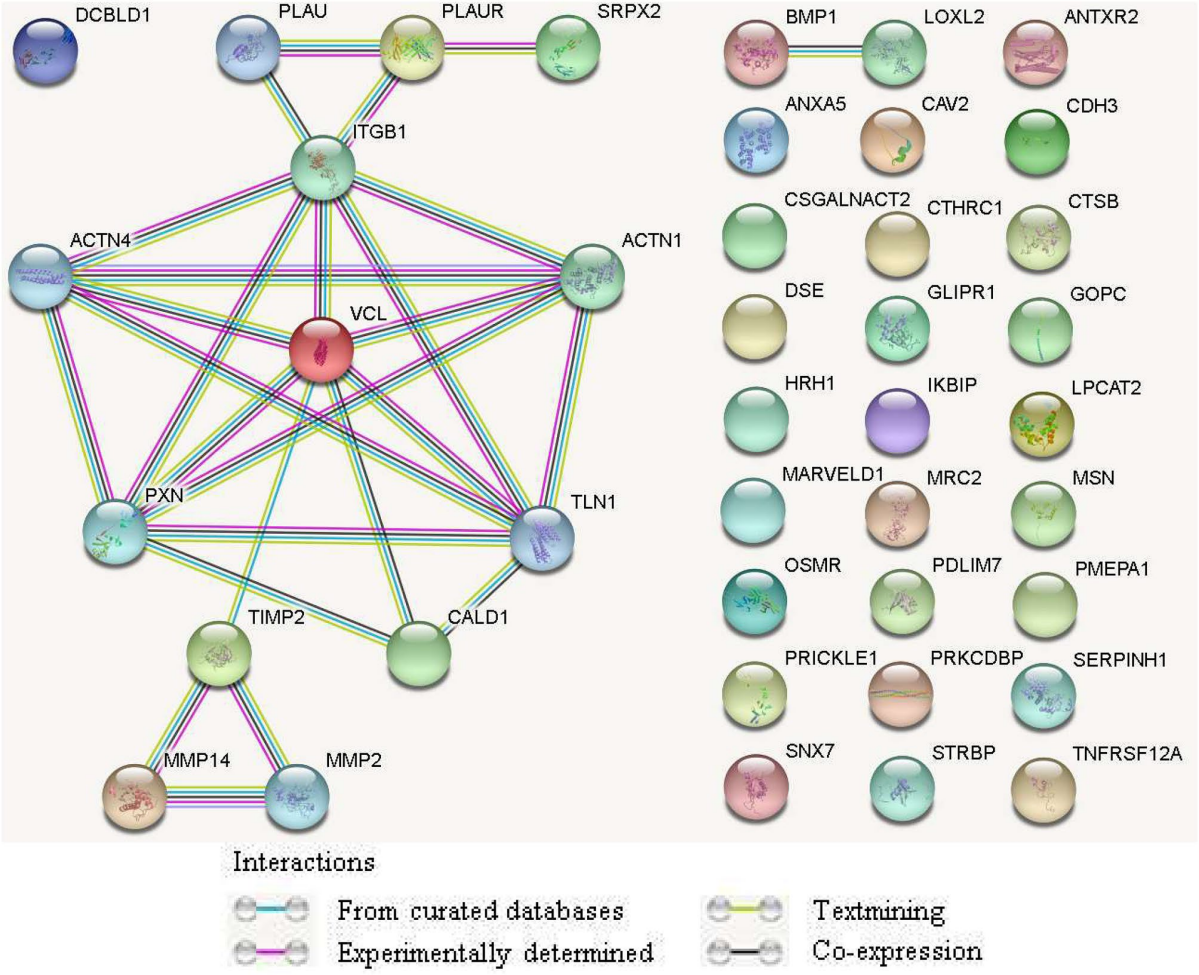
Differentially expressed genes in patients with high DCBLD1 expression NSCLC, invasive breast carcinoma and HNSCC. STRING protein interaction analysis of the 37 genes differentially regulated for patients with high *DCBLD1* expression (n = 50) in comparison to those with low *DCBLD1* expression (n = 50) in the TCGA cohorts for NSCLC, invasive breast carcinoma and HNSCC TCGA cohorts. The network shows results for the highest confidence interval (0.9) interaction scores on STRING v11 (https://string-db.org/). STRBP has lower expression, while the 36 other genes have higher expression in patients with high DCBLD1 expression.

There is three cancers for which high DCBLD1 expression has been associated to a worse overall survival and upregulation of the integrin signaling pathway: NSCLC, invasive breast carcinoma and HNSCC, which was previously published^2^. Evaluating the common changes in those three cancers for DCBLD1 high cases will allow to better understand DCBLD1 role in oncology and to further clarify how DCBLD1 is associated with the integrin signaling pathway. For this study, 37 common genes were differentially regulated between patients of high and low *DCBLD1* expression for NSCLC, invasive breast carcinoma and HNSCC. All these genes were upregulated in the high *DCBLD1* expression group with the exception of *STRBP*, which was downregulated. Interactions between these genes were evaluated using STRING protein interaction analysis with the highest confidence interval (0.9) (Fig. 4). This analysis allows to build the connectivity network of those genes for physical and functional interactions, using bioinformatics to combine publicly available sources of data^20^. A strong association was observed between *ITGB1*, *ACTN1*, *ACTN4*, *VCL*, *PXN*, *TLN1*, *PLAU*, *PLAUR* and *SRPX2*. An association between *TIMP2*, *MMP2* and *MMP14* was also observed. Other genes did not integrate in the network. DCBLD1 itself did not associate in the STRING connectivity network, although it was expected as its function is still undetermined. On the other hand, since DCBLD1 high expression is the common point for this analysis, it is likely that DCBLD1 should be inserted in those pathways. Further in vitro experiments will be necessary to determine where and if this association is physical or functional. For other genes which did not associate in the connectivity network, they are either unrelated to that network or their association has not yet been shown.

## Discussion

In this study, we showed that *DCBLD1* gene expression is prognostic of overall survival in NSCLC and breast cancer. For NSCLC and HNSCC, the association of germinal SNPs in the *DCBLD1* promoter region has been clearly established, especially for patients who are non-smokers or have no classical cancer risk factors^1–7^. Moreover, *DCBLD1* copy number alterations and mutations in the protein coding region are rare. This suggests that high *DCBLD1* expression in tumors may arise from SNPs in the promoter region modifying gene regulation or alterations in transcription factors, or both. These SNPs may have similar implications for invasive breast carcinoma, particularly for subtypes that are more likely to harbor germline mutations. Basal-like cancers are usually triple-negative breast cancers, which harbor more germline mutations of *BRCA1* and *BRCA2*^21,22^. In this study, we showed that basal-like cancers had a high expression of *DCBLD1* in comparison to other subtypes. Whether this involves germline SNPs in the *DCBLD1* promoter region is unknown and will need further study to determine an affiliation with this subtype.

In three cancers (NSCLC, invasive breast carcinoma and HNSCC) for which DCBLD1 had prognostic value, high *DCBLD1* expression showed statistically significant upregulation of the integrin signaling pathway. In contrast, high and low DCBLD1 expression showed no difference in the cancers for which *DCBLD1* had no prognostic value. We hypothesized that an oncogenic role for *DCBLD1* was associated with the activation of the integrin signaling pathway.

Using STRING protein interaction analysis, the upregulated genes in patients with high *DCBLD1* expression revealed an important network of nine proteins that centered on ITGB1. ITGB1 is a transmembrane integrin that interacts with the ECM and stimulates cell–matrix adhesion when bound to a phosphorylated ACTN1^23,24^. VCL, PXN and TLN1 are adapter proteins that bind to ITGB1 and ACTN1, forming a link between ITGB1 and actin filaments^25,26^. These five proteins are central components of focal adhesions, which allow the intracellular actin cytoskeleton to associate with the ECM^27,28^. The reminder of the nine identified proteins includes ACTN4, which shares 86.7% amino acid sequence with ACTN1 and also binds to ITGB1, but its role in regulating focal adhesions is less clear^29^. PLAU and its receptor PLAUR are involved in the proteolysis of the ECM and mediate cleavage of ITGA6, which forms the heterodimeric laminin receptor with ITGB1^30,31^. SRPX2 is another PLAUR ligand^32^, but its association with focal adhesions is unclear. Lastly, TIMP2, MMP2 and MMP14 are mediators of ECM degradation associated with tumor metastasis^33^. For NSCLC, invasive breast carcinoma and HNSCC, the upregulation of all these proteins in conjunction with high *DCBLD1* expression strongly suggests that DCBLD1 is involved in focal adhesions and therefore, cell migration.

Previous in vitro experiments reveal that the DCBLD1 interactome consists mainly of adaptor proteins and proteins associated with actin dynamics^15^, further implicating a role for DCBLD1 in focal adhesions and supporting the idea that DCBLD1 is a NRP-like protein. Both NRP1 and NRP2 are involved in focal adhesions: NRP1 regulates focal adhesion turnover and NRP2 regulates α6β1 integrin association with the cytoskeleton^11,34^. Although the extracellular domains of DCBLD1 and NRP are very similar, their intracellular domains are completely different. NRP1 and NRP2 have small intracellular domains of 44 and 42 amino acids, respectively, whereas DCBLD1 has a more complex intracellular domain of 235 amino acids with multiple YxxP motifs^8,14^. The exact role of DCBLD1 in focal adhesion formation is yet to be discovered, but the role of focal adhesion turnover in tumor cell migration has already been established^27^ and may provide insight into the poor prognosis among potentially aggressive cancers with high *DCBLD1* expression. Upregulation of the integrin signaling pathway was not observed for colorectal and prostate cancers, the prognosis was similar for patients with high or low *DCBLD1* expression within these cohorts, suggesting that DCBLD1 activity is cell-type dependent. On the other hand, we also showed that *DCBLD1* expression is higher in tumor tissue for all four cancers. The fact that *DCBLD1* was upregulated also in cancers for which it has no prognostic value suggests that factors regulating DCBLD1 might be generally regulated in cancer. Identifying how DCBLD1 gene expression is regulated might help understand why it has a prognostic value in some cancers.

Association between cancer migration and patient survival is well established, and more specifically for breast cancer, a migration transcriptomic signature was previously published and showed to predict overall survival for that cohort^35^. We hypothesize that DCBLD1 expression prognostic value also comes from DCBLD1 association with migration through upregulation of the integrin signaling pathway and perhaps more importantly regulation of focal adhesion. Since one study evaluating the oncological role of DCBLD1 using the A549 lung adenocarcinoma cell line showed a decrease in xenograft tumor growth when using a stable DCBLD1 knockdown cell line^7^, it is reasonable to hypothesize that DCBLD1 has a regulating role in those pathways. This also suggests that DCBLD1 could be a potential therapeutic target.

The retrospective design of this study was the main limitation of the study and may have introduced bias and confounding factors. We used multivariate analysis when examining patient outcome to overcome this limitation as much as possible, although it is likely that some confounding factors were not included in the multivariate analysis. To limit censor bias, we used overall survival and biochemical recurrence as outcomes as they are well defined. Also outcome was evaluated until 95% of the patients were either censored or deceased to prevent potential bias from some rare patients with very long follow-up. Immortal time bias was prevented because samples were taken at surgery (day 0) in those cohorts, on the same specimen as pathology assessment. Another limitation was that analyses focused on RNA expression data and not actual protein levels. The prognostic value of measured DCBLD1 protein levels in NSCLC, invasive breast carcinoma and HNSCC will warrant further studies.

## Conclusion

Using multiple cancer cohorts, this study showed that *DBCLD1* is associated with the integrin signaling pathway and focal adhesions, and has prognostic value for NSCLC and invasive breast carcinoma. Given that germline SNPs in *DCBLD1* are associated with non-smoking lung and head and neck cancers, and demonstrate prognostic value in other cancers, further studies are needed to evaluate its potential as a therapeutic target.

## Materials and methods

### Study cohorts

This retrospective study included multiple independent cohorts. NSCLC was represented by the NSCLC TCGA cohort, which combined the TCGA Firehose Legacy LUAD^36^ and the TCGA Firehose Legacy Lung Squamous Cell Carcinoma (LSCC)^37^ cohorts, and by the GSE81089 NSCLC^38^ cohort. For invasive breast carcinoma, the TCGA Firehose Legacy Breast Invasive Carcinoma^39^ and the METABRIC^40^ cohorts were investigated. For colorectal adenocarcinoma, the TCGA Firehose Legacy Colorectal Adenocarcinoma^41^ and the GSE14333^42^ cohorts were analyzed. For prostate adenocarcinoma, the TCGA Firehose Legacy Prostate Adenocarcinoma^43^ and the GSE70770^44,45^ cohorts were examined. For HNSCC, the TCGA provisional cohort^46^ was used.

Clinical data from the TCGA and METABRIC cohorts were obtained using cBioPortal for Cancer Genomics (www.cbioportal.com)^47,48^. TCGA mRNAseq data was extracted from FirehoseR (gdac.broadinstitute.org)^49^. Data from the GSE14333, GSE81089 and GSE70770 cohorts were extracted from the National Center for Biotechnology Information (NCBI) Gene Expression Omnibus database^50^. Every participant of these cohorts with RNA expression and outcome data were included in the study. Analysis of the TCGA PanCancer Atlas^51^ was performed with the cBioPortal^47,48^. No patient was excluded for this analysis. The data for upregulated and downregulated genes for HNSCC was previously published^2^.

### Clinical characteristics

*DCBLD1* gene expression was measured by RNA-seq for all TCGA cohorts and GSE81089, and measured by array for the METABRIC, GSE14333 and GSE70770 cohorts. *DCBLD1* gene expression was normalized using z-score normalization of the log expression value, except for tumor versus normal adjacent tissue comparison where it was analyzed as log_2_ RSEM.

For multivariate analysis, *DCBLD1* gene expression and age were analyzed as numerical variables. Stages were subdivided into two groups: stage 1 and 2 versus stage 3 and 4. Patients were subdivided by sex when possible except for invasive breast carcinoma, which had only 12 males in the TCGA cohort and no males in the METABRIC cohort. Outcome was evaluated until 95% of the patients were either censored or deceased to prevent potential bias from some rare patients with very long follow-up. Samples were taken at surgery (day 0) in those cohorts, on the same specimen as pathology assessment. For NSCLC, tobacco use (ever versus never users) and histology (LUAD versus LSCC) were also assessed. For invasive breast carcinoma, PAM50 subtypes were also evaluated. For prostate adenocarcinoma, biochemical recurrence was used.

### Statistics

HR was evaluated using the multivariate Cox proportional hazards analysis for the multivariate survival prediction model. The significance of the gene expression variations was determined by Student’s *t*-test and Tukey’s honest significance test for nominal variables. *DBCLD1* gene expression and age association was evaluated using linear regression. *DCBLD1* gene expression comparison for paired normal and tumor tissue was done using a paired Student’s *t*-test. Pathway enrichment was evaluated using the PANTHER pathway database^52^. For the PANTHER pathways annotation data set, Fisher’s exact test was corrected using FDR. Interactions between proteins was determined using STRING v11 (https://string-db.org/) with the highest confidence threshold (0.9)^20^.

Tests of statistical significance were two-sided and *P* values less than 0.05 were considered significant with *P < 0.05, **P < 0.01 and ***P < 0.001. Statistical analysis was performed using JMP 12.0.1 statistical software (SAS Institute Inc) with the exception of the whole exome analysis, which was performed using GraphPad Prism 7.04 (GraphPad Software Inc.).

## Supplementary Information

Supplementary Information.

## Abbreviations

CI: Confidence interval
ECM: Extracellular matrix
FDR: False discovery rate
HNSCC: Head and neck squamous cell carcinoma
HR: Hazards ratio
LSCC: Lung squamous cell carcinoma
LUAD: Lung adenocarcinoma
METABRIC: Molecular Taxonomy of Breast Cancer International Consortium
NCBI: National Center for Biotechnology Information
NRP: Neuropilin
NSCLC: Non-small cell lung carcinoma
SNP: Single nucleotide polymorphism
TCGA: The Cancer Genome Atlas

## References

[1] Lan Q (2012). Genome-wide association analysis identifies new lung cancer susceptibility loci in never-smoking women in Asia. Nat. Genet..

[2] Cardin GB (2019). Single nucleotide polymorphism rs6942067 is a risk factor in young and in non-smoking patients with HPV negative head and neck squamous cell carcinoma. Cancers (Basel)..

[3] Hung RJ (2019). Lung cancer risk in never-smokers of european descent is associated with genetic variation in the 5p15.33 TERT-CLPTM1Ll region. J. Thorac. Oncol..

[4] Yoo SS (2017). Effects of polymorphisms identified in genome-wide association studies of never-smoking females on the prognosis of non-small cell lung cancer. Cancer Genet..

[5] Yang C (2018). Positional integration of lung adenocarcinoma susceptibility loci with primary human alveolar epithelial cell epigenomes. Epigenomics.

[6] Han JF (2016). Polymorphism of rs9387478 correlates with overall survival in female nonsmoking patients with lung cancer. Int. J. Biol. Markers.

[7] Wang Y (2020). SNP rs17079281 decreases lung cancer risk through creating an YY1-binding site to suppress DCBLD1 expression. Oncogene.

[8] UniProt Consortium (2019). UniProt: A worldwide hub of protein knowledge. Nucleic Acids Res..

[9] Nartey MN (2020). Learning-induced mRNA alterations in olfactory bulb mitral cells in neonatal rats. Learn. Mem..

[10] Goel HL, Mercurio AM (2012). Enhancing integrin function by VEGF/neuropilin signaling: implications for tumor biology. Cell Adh. Migr..

[11] Goel HL, Pursell B, Standley C, Fogarty K, Mercurio AM (2012). Neuropilin-2 regulates alpha6beta1 integrin in the formation of focal adhesions and signaling. J. Cell Sci..

[12] Valdembri D (2009). Neuropilin-1/GIPC1 signaling regulates alpha5beta1 integrin traffic and function in endothelial cells. PLoS Biol..

[13] Cao Y (2013). Neuropilin-2 promotes extravasation and metastasis by interacting with endothelial alpha5 integrin. Cancer Res..

[14] Schmoker AM (2017). Dynamic multi-site phosphorylation by Fyn and Abl drives the interaction between CRKL and the novel scaffolding receptors DCBLD1 and DCBLD2. Biochem. J..

[15] Schmoker AM (2020). FYN and ABL regulate the interaction networks of the DCBLD receptor family. Mol.. Cell Proteomics.

[16] Siegel RL, Miller KD, Jemal A (2020). Cancer statistics, 2020. CA Cancer J. Clin..

[17] Ranganathan P, Pramesh CS, Aggarwal R (2017). Common pitfalls in statistical analysis: Logistic regression. Perspect. Clin. Res..

[18] Caan BJ (2014). Intrinsic subtypes from the PAM50 gene expression assay in a population-based breast cancer survivor cohort: Prognostication of short- and long-term outcomes. Cancer Epidemiol. Biomark. Prev..

[19] Lin X (2019). Assessment of biochemical recurrence of prostate cancer (Review). Int. J. Oncol..

[20] Szklarczyk D (2019). STRING v11: protein-protein association networks with increased coverage, supporting functional discovery in genome-wide experimental datasets. Nucleic Acids Res..

[21] Gonzalez-Angulo AM (2011). Incidence and outcome of BRCA mutations in unselected patients with triple receptor-negative breast cancer. Clin. Cancer Res..

[22] Hartman AR (2012). Prevalence of BRCA mutations in an unselected population of triple-negative breast cancer. Cancer.

[23] Craig DH, Haimovich B, Basson MD (2007). Alpha-actinin-1 phosphorylation modulates pressure-induced colon cancer cell adhesion through regulation of focal adhesion kinase-Src interaction. Am. J. Physiol. Cell Physiol..

[24] Shenoy PS (2001). beta1 Integrin-extracellular matrix protein interaction modulates the migratory response to chemokine stimulation. Biochem. Cell Biol..

[25] Otey CA, Pavalko FM, Burridge K (1990). An interaction between alpha-actinin and the beta 1 integrin subunit in vitro. J. Cell Biol..

[26] Schaller MD, Otey CA, Hildebrand JD, Parsons JT (1995). Focal adhesion kinase and paxillin bind to peptides mimicking beta integrin cytoplasmic domains. J. Cell Biol..

[27] Nagano M, Hoshino D, Koshikawa N, Akizawa T, Seiki M (2012). Turnover of focal adhesions and cancer cell migration. Int. J. Cell Biol..

[28] Bois PR, Borgon RA, Vonrhein C, Izard T (2005). Structural dynamics of alpha-actinin–vinculin interactions. Mol. Cell Biol..

[29] Pavalko FM, Otey CA, Simon KO, Burridge K (1991). Alpha-actinin: a direct link between actin and integrins. Biochem. Soc. Trans..

[30] Smith HW, Marshall CJ (2010). Regulation of cell signalling by uPAR. Nat. Rev. Mol. Cell Biol..

[31] Demetriou MC, Pennington ME, Nagle RB, Cress AE (2004). Extracellular alpha 6 integrin cleavage by urokinase-type plasminogen activator in human prostate cancer. Exp. Cell Res..

[32] Bruneau N, Szepetowski P (2011). The role of the urokinase receptor in epilepsy, in disorders of language, cognition, communication and behavior, and in the central nervous system. Curr. Pharm. Des..

[33] Shen Q, Lee ES, Pitts RL, Wu MH, Yuan SY (2010). Tissue inhibitor of metalloproteinase-2 regulates matrix metalloproteinase-2-mediated endothelial barrier dysfunction and breast cancer cell transmigration through lung microvascular endothelial cells. Mol. Cancer Res..

[34] Seerapu HR (2013). The cytoplasmic domain of neuropilin-1 regulates focal adhesion turnover. FEBS Lett..

[35] Nair NU (2019). Migration rather than proliferation transcriptomic signatures are strongly associated with breast cancer patient survival. Sci. Rep..

[36] Cancer Genome Atlas Research Network (2014). Comprehensive molecular profiling of lung adenocarcinoma. Nature.

[37] Cancer Genome Atlas Research Network (2012). Comprehensive genomic characterization of squamous cell lung cancers. Nature.

[38] Mezheyeuski A (2018). Multispectral imaging for quantitative and compartment-specific immune infiltrates reveals distinct immune profiles that classify lung cancer patients. J. Pathol..

[39] Network CGA (2012). Comprehensive molecular portraits of human breast tumours. Nature.

[40] Curtis C (2012). The genomic and transcriptomic architecture of 2,000 breast tumours reveals novel subgroups. Nature.

[41] Network CGA (2012). Comprehensive molecular characterization of human colon and rectal cancer. Nature.

[42] Jorissen RN (2009). Metastasis-associated gene expression changes predict poor outcomes in patients with dukes stage B and C colorectal cancer. Clin. Cancer Res..

[43] Cancer Genome Atlas Research Network (2015). The molecular taxonomy of primary prostate cancer. Cell.

[44] Ross-Adams H (2015). Integration of copy number and transcriptomics provides risk stratification in prostate cancer: A discovery and validation cohort study. EBioMedicine.

[45] Whitington T (2016). Gene regulatory mechanisms underpinning prostate cancer susceptibility. Nat. Genet..

[46] Network CGA (2015). Comprehensive genomic characterization of head and neck squamous cell carcinomas. Nature.

[47] Cerami E (2012). The cBio cancer genomics portal: an open platform for exploring multidimensional cancer genomics data. Cancer Discov..

[48] Gao J (2013). Integrative analysis of complex cancer genomics and clinical profiles using the cBioPortal. Sci. Signal.

[49] Deng, M., Bragelmann, J., Kryukov, I., Saraiva-Agostinho, N. & Perner, S. FirebrowseR: An R client to the Broad Institute's Firehose Pipeline. *Database (Oxford)* **2017**, 10.1093/database/baw160 (2017). 10.1093/database/baw160 PMC5216271 28062517

[50] Edgar R, Domrachev M, Lash AE (2002). Gene Expression Omnibus: NCBI gene expression and hybridization array data repository. Nucleic Acids Res..

[51] Cancer Genome Atlas Research Network. *et al.* The Cancer Genome Atlas Pan-Cancer analysis project. *Nat. Genet.* **45**, 1113–1120, doi:10.1038/ng.2764 (2013). 10.1038/ng.2764 PMC3919969 24071849

[52] Mi H, Muruganujan A, Ebert D, Huang X, Thomas PD (2019). PANTHER version 14: more genomes, a new PANTHER GO-slim and improvements in enrichment analysis tools. Nucleic Acids Res..

