# Supplementary material for: DCBLD1 is associated with the integrin signaling pathway and has prognostic value in non-small cell lung and invasive breast carcinoma
Source: Sci Rep. 2021 Jun 17;11:12753. doi: 10.1038/s41598-021-92090-6 (PMC8211811; doi:10.1038/s41598-021-92090-6)
Supplement: Supplementary file 1 — Supplementary Information. [file 41598_2021_92090_MOESM1_ESM.pdf]

Supplementary Information

Title: DCBLD1 is associated with the integrin signaling pathway and has prognostic value in non-small cell lung and invasive breast carcinoma

Authors and affiliations: Guillaume B. Cardin[1,2], Monique Bernard[1,2], Francis Rodier[1,2,3], and Apostolos Christopoulos[1,2,4*].

[1] Centre de recherche du Centre hospitalier de l'Université de Montréal, Montreal, QC, Canada.

[2] Institut du cancer de Montréal, Montreal, QC, Canada.

[3] Département de radiologie, radio-oncologie et médecine nucléaire, Université de Montréal, Montreal, QC, Canada.

[4] Otolaryngology-Head and Neck Surgery Service, Centre hospitalier de l'Université de Montréal, Montreal, QC, Canada.

Supplmentary Table 1. Multivariate Cox proportional hazards analysis of NSCLC outcomes

Variable	HR (95% CI)	*P*	HR (95% CI)	*P*
**Non-small cell lung carcinoma – overall survival**				
	**TCGA**		**GSE81089**	
DCBLD1[a]	3.19 (1.15-8.60)	0.03	4.34 (1.10-17.36)	0.04
Age[a]	2.15 (1.17-3.98)	0.01	1.18 (0.40-3.55)	0.77
Sex[b]	1.16 (0.90-1.42)	0.29	1.18 (0.75-1.87)	0.48
Stage (2 vs 1)	1.56 (1.21-2.00)	<0.0001	1.64 (0.93-2.85)	0.09
Stage (3 vs 1)	2.18 (1.66-2.84)	<0.0001	---	---
Stage (4 vs 1)	3.15 (1.92-4.92)	<0.0001	---	---
Stage (3-4 vs 1)[c]	---	---	3.15 (1.81-5.41)	<0.0001
Tobacco use[d]	0.90 (0.62-1.35)	0.6	1.95 (0.85-5.62)	0.12
Histology[e]	1.01 (0.82-1.30)	0.78	0.94 (0.57-1.52)	0.79

[a] DCBLD1 and age hazard ratios are calculated using the data as a continuous variable.

[b] Sex hazard ratio is calculated as the risk of being male versus the risk of being female.

[c] There is only three stage 4 patients in the GSE81089 cohort,

therefore stage 3 and 4 were pooled for the analysis of that cohort

[d] Tobacco use hazard ratio is calculated as the risk of ever smoker versus never smoker.

[e] Histology hazard ratio is calculated as the risk of LSCC versus LUAD

Supplementary Table 2. Multivariate Cox proportional hazards analysis of invasive breast carcinoma outcomes

**Variable**	**HR (95% CI)**	***P***	**HR (95% CI)**	***P***
**Invasive breast carcinoma – overall survival**				
	**TCGA**		**METABRIC**	
DCBLD1[a]	9.47 (0.96-84.53)	0.05	1.80 (1.11-2.94)	0.02
Age[a]	8.61 (1.17-3.98)	<0.0001	12.71 (7.86-20.55)	<0.0001
Stage[c]	3.48 (2.30-5.25)	<0.0001	2.19 (1.75-2.70)	<0.0001
PAM50 (normal vs LumA)	1.23 (0.30-3.41)	0.74	1.23 (0.97-1.79)	0.09
PAM50 (LumB vs LumA)	1.56 (0.92-2.61)	0.10	1.30 (1.08-1.56)	0.006
PAM50 (basal vs LumA)	1.61 (0.86-2.92)	0.13	1.34 (0.97-1.79)	0.07
PAM50 (Her2 vs LumA)	2.96 (1.50-5.51)	0.003	1.67 (1.31-2.11)	<0.0001

[a] DCBLD1 and age hazard ratios are calculated using the data as a continuous variable.

[b] Sex hazard ratio is calculated as the risk of being male versus the risk of being female.

[c] Stage hazard ratio is calculated as the risk of higher stage (3 or 4) versus the risk of lower stage (1 and 2).

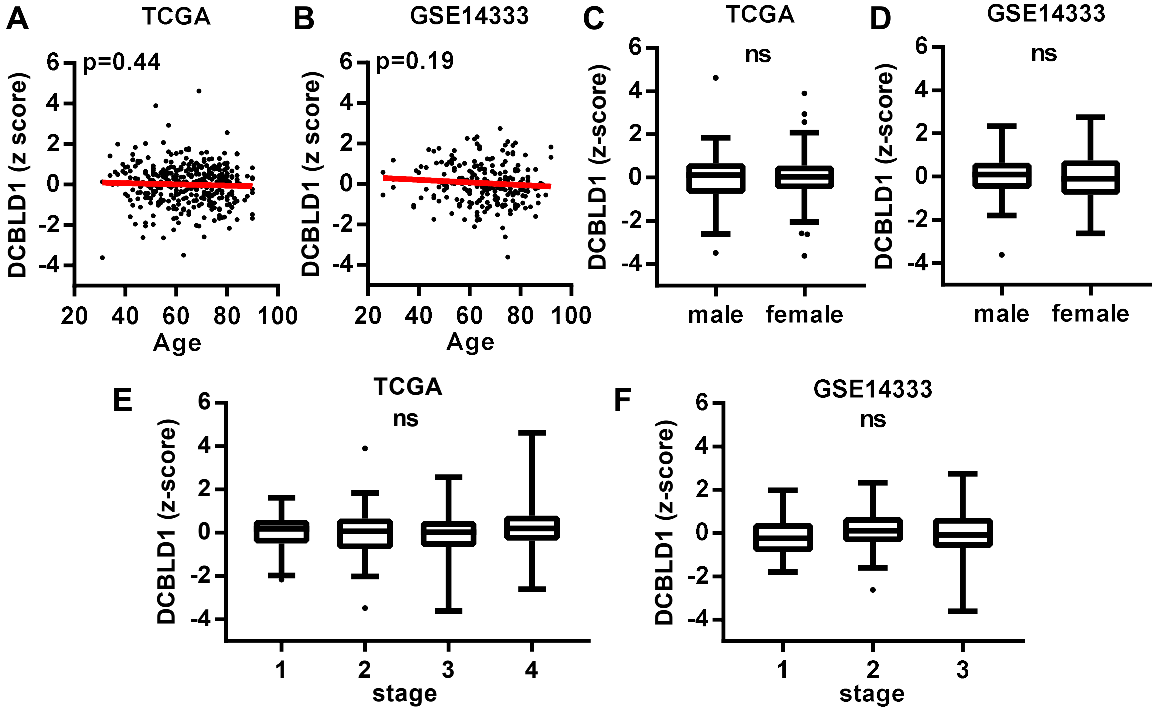

Supplementary Figure 1. DCBLD1 in the DCBLD1 in the TCGA and GSE14333 colorectal adenocarcinoma cohorts. Comparison of DCBLD1 gene expression for age (**A** and **B**), sex (**C** and **D**) and stage (**E** and **F**).and GSE14333 colorectal adenocarcinoma cohorts.

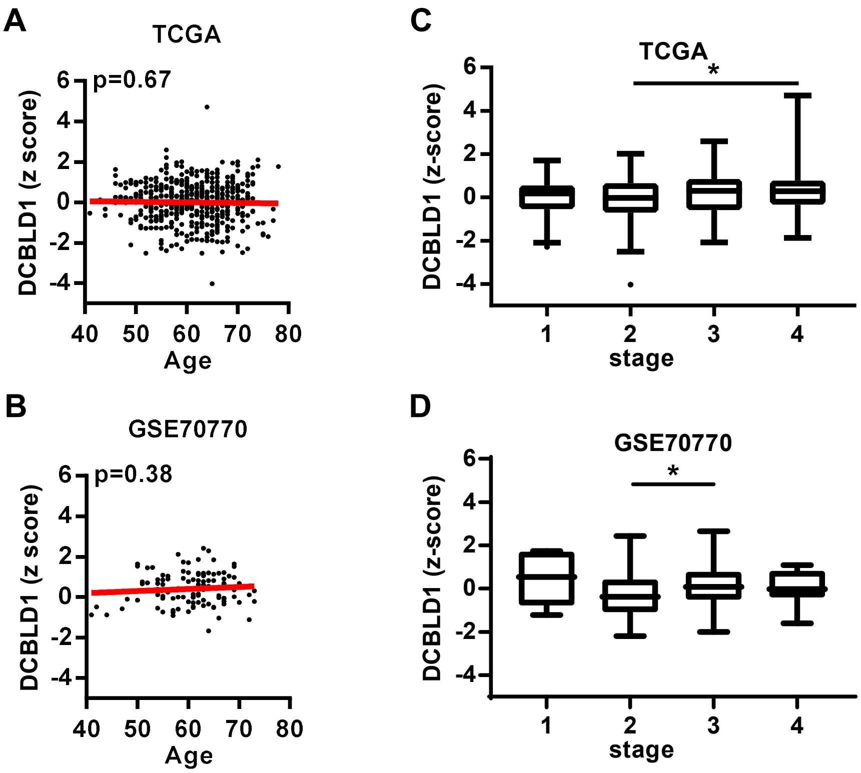

Supplementary Figure 2. DCBLD1 in the TCGA and GSE70770 prostate adenocarcinoma cohorts. Comparison of DCBLD1 gene expression for age (**A** and **B**) and stage (**C** and **D**).

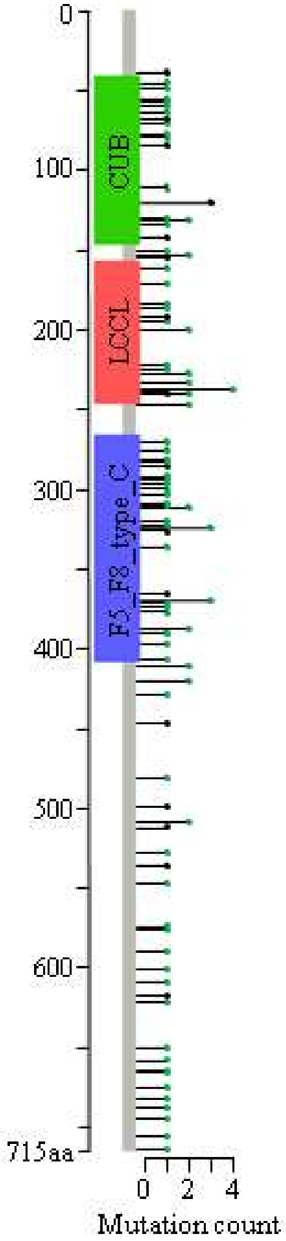

Supplementary Figure 3. *DCBLD1* mutations in the TCGA PanCancer Atlas studies. Modified from https://www.cbioportal.org/

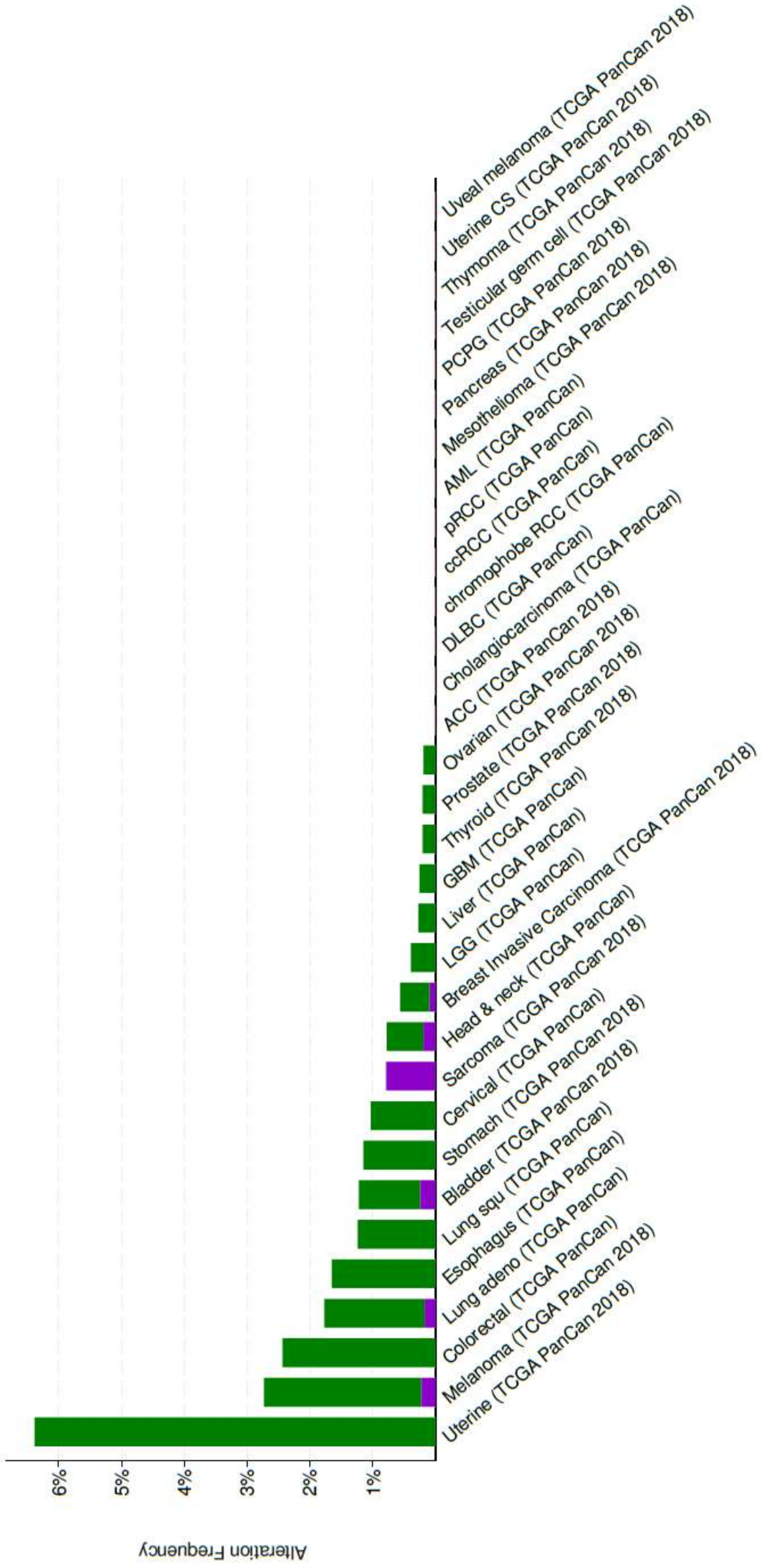

Supplementary Figure 4. DCBLD1 mutation and gene fusion occurrence in the TCGA PanCancer Atlas. From https://www.cbioportal.org/

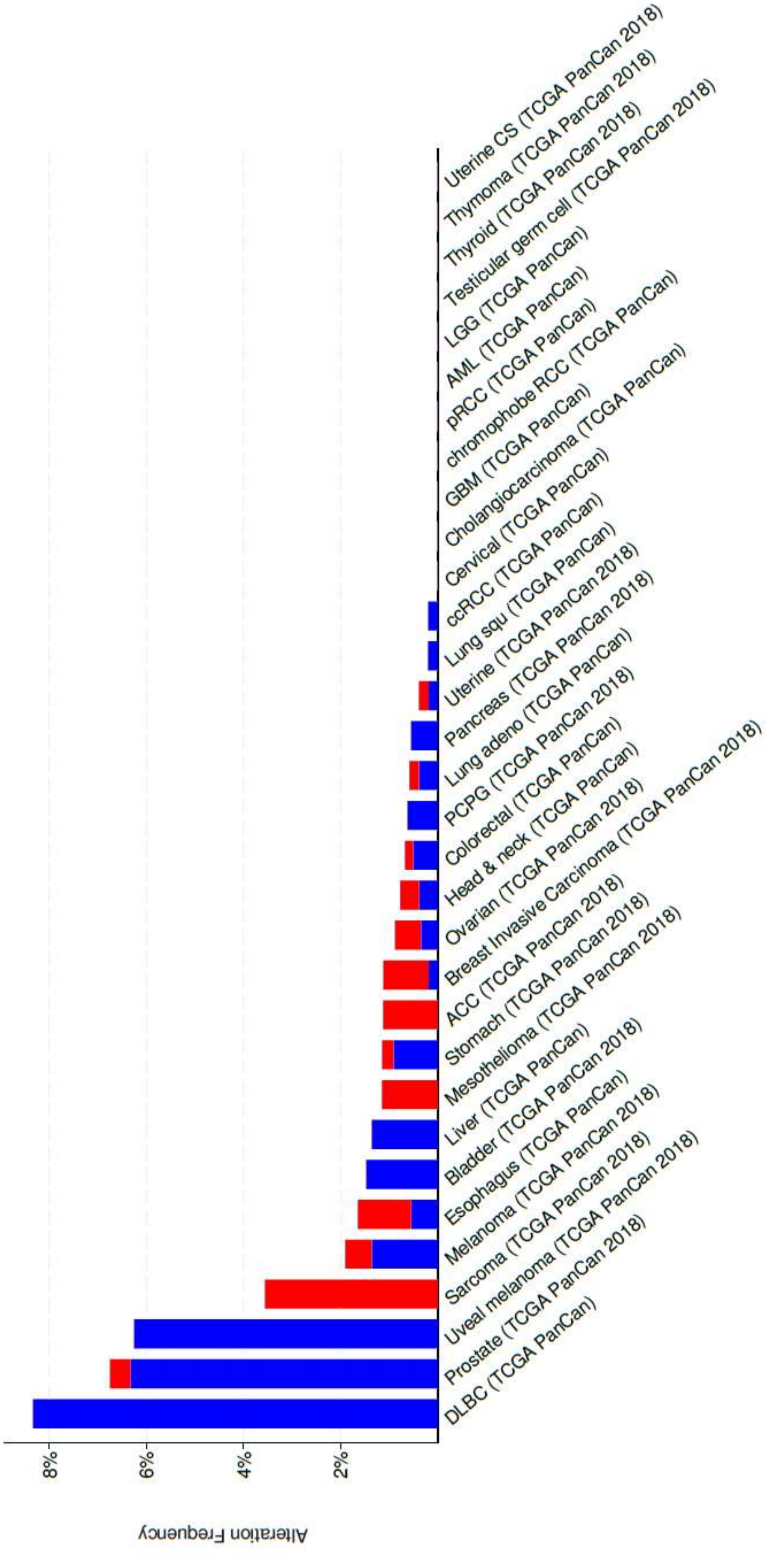

Supplementary Figure 5. DCBLD1 copy number alterations occurrence in the TCGA PanCancer Atlas. From https://www.cbioportal.org/
